# Medicinal Plants from Near East for Cancer Therapy

**DOI:** 10.3389/fphar.2018.00056

**Published:** 2018-01-31

**Authors:** Mohammad S. Abu-Darwish, Thomas Efferth

**Affiliations:** ^1^Department of Basic and Applied Sciences, Shoubak University College, Al-Balqa’ Applied University, Al-Salt, Jordan; ^2^Department of Pharmaceutical Biology, Institute of Pharmacy and Biochemistry, Johannes Gutenberg University, Mainz, Germany

**Keywords:** complementary and alternative medicine, pharmacognosy, phytochemistry, traditional medicine, Near East, cancer

## Abstract

**Background:** Cancer is one of the major problems affecting public health worldwide. As other cultures, the populations of the Near East rely on medicinal herbs and their preparations to fight cancer.

**Methods:** We compiled data derived from historical ethnopharmacological information as well as *in vitro* and *in vivo* results and clinical findings extracted from different literature databases including (PubMed, Scopus, Web of Science, and Google Scholar) during the past two decades.

**Results:** In this survey, we analyzed the huge amount of data available on anticancer ethnopharmacological sources used in the Near East. Medicinal herbs are the most dominant ethnopharmacological formula used among cancer’s patients in the Near East. The data obtained highlight for the first time the most commonly used medicinal plants in the Near East area for cancer treatment illustrating their importance as natural anticancer agents. The literature survey reveals that various *Arum* species, various *Artemisia* species, *Calotropis procera*, *Citrullus colocynthis*, *Nigella sativa*, *Pulicaria crispa*, various *Urtica* species, *Withania somnifera*, and others belong to the most frequently used plants among cancer patients in the Near East countries. Molecular modes of action that have been investigated for plant extracts and isolated compounds from Near East include cell cycle arrest and apoptosis induction with participation of major player in these processes such as p53 and p21, Bcl-2, Bax, cytochrome *c* release, poly (ADP-ribose) polymerase cleavage, activation of caspases, etc.

**Conclusion:** The ethnopharmacology of the Near East was influenced by Arabic and Islamic medicine and might be promising for developing new natural and safe anticancer agents. Further research is required to elucidate their cellular and molecular mechanisms and to estimate their clinical activity.

## Introduction

### Flora and Medicinal Plants of the Near East

The Near East region with its long-lasting history comprises the countries of the Arabian Peninsula, Egypt, Iraq, Iran, Israel, Jordan, Lebanon, Palestinian territories, Syria, and Turkey according to the National Geographic Society world map (Frodin, 2001; National Geographic Society (US), 2009). The territory of the Arabian Peninsula spans a large area of the Near East region, including Saudi Arabia, Yemen (including Socotra), Oman, United Arab Emirates, Qatar, Bahrain, and Kuwait. It is surrounded by the Arabian Sea in the east and the Red Sea in the west. It is also surrounded by Syria, Jordan, and Iraq in the north and by the Indian Ocean in the south (Ash, 2005).

Worldwide, 50,000–80,000 flowering plants are used for medicinal and therapeutic purposes (Marinelli, 2005). The Flora of the Near East region is highly diverse and comprises 23,000 vascular plant species, which of 6,700 are endemic (Boulos et al., 1994; Heywood, 2004).

The flora of Arabian Peninsula comprises 7,801 vascular plant species of which 509 are endemic (Boulos et al., 1994). A total of 2,250 species belonging to 142 families have been recorded in the Saudi Arabian flora (Collenette, 1998). Among them, there are 242 endemic and 600 rare and endangered species.

The flora of Bahrain, Kuwait, Oman, Qatar, United Arab Emirates (UAE), and Yemen (including Socotra) comprises about 248, 282, 1,200, 306, 340, and 3,640 vascular plant species, respectively (Abbas et al., 1992; Saganuwan, 2010; Hall and Miller, 2011; Sakkir et al., 2012). Approximately 11% of the species are endemic or regionally endemic (Boulos et al., 1994; Saganuwan, 2010). With exception of Yemen, where no data is available, the numbers of plant species categorized as medicinal in the countries of Arabian Peninsula are listed in **Table 1**.

**Table 1:** Plant species with medicinal use in countries of the Arabian Peninsula.

Country	Number of medicinal plants	Reference
Bahrain	52	Abbas et al., 1992
Oman	485	Ghazanfar and Al-Al-Sabahi, 1993
Qatar	184	Rizk and El-Ghazaly, 1995
Saudi Arabia	1,200	Rahman et al., 2004
UAE	20% of all species in UAE are used in folk medicine	Sakkir et al., 2012


Israel, Jordan, Lebanon, Palestinian territories, and Syria are located in the geographical region that is historically known as Bilad all-Sham. This region comprises about 4,500 species (Zohary, 1983; Saganuwan, 2010; Al-Eisawi, 2013).

Israel and Palestinian territories have 2,600 plant species belonging to 130 different families (Zohary, 1983). Many of these plants are native and have medicinal value in the traditional medicines of the Bedouin, Druze, Galilee Arabs, and Middle Eastern Jewish communities (Lev, 2006). An updated checklist of the Jordanian flora recorded 2,543 species, 868 genera and 142 families in Jordan (Al-Eisawi, 2013). Among them, 363 species of vascular plants belonging to 263 genera and 86 families have documented uses in traditional medicine (Oran and Al-Eisawi, 1998; Abu-Darwish et al., 2014).

Lebanon and Syria have the richest flora of the Bilad all-Sham region. Lebanon has 2,607 species, with 783 genera, which of them 78 are endemic (Nehme, 1978). In Syria, 3,500 vascular plants belonging to 865 genera and 131 families have been recorded (Wahbe, 1999). More than 130 plant species of the Lebanese flora are used in the folk medicine (Abu Chaar, 2004; Deeb et al., 2013; Baydoun et al., 2015). Egypt (including Sinai) and Iraq have the richest plant diversity with 3,005 and 3,000 vascular plant species, respectively (Boulos et al., 1994).

Iran and Turkey are the Near East countries with the richest biodiversity. They have 8,000 and 8,650 species, respectively (Boulos et al., 1994). Among them, about 1,100 species are used in Iranian folk medicine (Mashayekhan et al., 2016), and 500–1,000 plant taxa are used in traditional Turkish medicine (Öztürk et al., 2012).

### Cancer Epidemiology in the Near East

Cancer is a major public health problem with significant death rates. Ten million cases were diagnosed in 1996, and the number of diagnosed cases in 2020 is estimated to increase to 20 million. With some probability, cancer will become the leading cause of death worldwide, exceeding the combined death rates AIDS, tuberculosis, and malaria (Salim et al., 2009). In developing countries including most countries of the Near East, cancer belongs to the three leading causes of death (Huerta and Grey, 2007; Salim et al., 2009). In the Arabian world, carcinoma of the lung, liver, or bladder cancers are most common among men, and breast cancer is most common among women (Makhoul and El-Barbir, 2006; Salim et al., 2009).

The Gulf Center for Cancer Registration (GCCR) reported that there were 95,183 newly diagnosed cancer cases among nationals of the countries of the Gulf Cooperation Council (GCC) including UAE, Bahrain, Saudi Arabia, Oman, Qatar, and Kuwait between the years 1998 and 2007. Non-Hodgkin lymphoma (NHL) was the predominant cancer entity among males and contributed to 8.8% of the total cancers, followed by leukemia and cancers of the colorectum, lung, and liver. In females, breast cancer was the commonest cancer, which accounted for 23.5% of all cancers followed by the thyroid and colorectal carcinoma, NHL, and leukemia (Al-Madouj et al., 2011).

Several reports showed the cancer incidence in the other countries of the Near East. In Cyprus, Egypt, Israel (Jews and Arabs), and Jordan the overall cancer incidence was substantially higher in the Jewish Israeli population than in the other populations of these countries for the time period of 1996–2001. They had the highest rate of colorectal cancer (Freedman et al., 2006). The patterns of high cancer incidence among the populations of the Near East countries are summarized in **Table 2**.

**Table 2:** Patterns of high cancer incidence among the populations of Near East countries.

Country(ies)	Cancer with high incidence	Reference
The countries of the GCC	In male: NHL followed by leukemia and cancers of colorectum, lung, and liver.	Al-Madouj et al., 2011
	In female: Breast cancer followed by thyroid and colorectal carcinoma, NHL, and leukemia.	
Egypt, Israel (Jews and Arabs), and Jordan	Cancer of the digestive system accounted for 20% of all cancers and breast cancer for about one-third of female cancers.	Freedman et al., 2006
	Colorectal cancer has the highest incidence among Israeli Jewish population.	
Lebanon	In male: Prostate cancer followed by bladder and lung cancer.	Shamseddine et al., 2014
	In female: Breast cancer followed by colon and lung carcinoma.	
Syria	Population (age of 20–39 years): Breast cancer followed by digestive system cancer and lymphoma.	Deeb and Eid, 2012
	Population (age of 40–59 years): Digestive system and gynecological cancers.	
Iraq	Laryngeal and urinary bladder cancers.	Salim et al., 2009
Turkey	Lung cancer followed by prostate, skin, breast and stomach cancer.	Yılmaz et al., 2011
Iran	Breast and stomach cancer	Mousavi et al., 2009


## Anticancer Ethnopharmacology of the Near East

Native herbal medicine is the basis of the Materia Medica of the entire Near East region (Batanouny, 1981; Al-Easa et al., 1990; Rizk and Heiba, 1990; Abbas et al., 1992). The nations in Near East share common cultural systems. Their ancient traditional medicines influenced each other by experience. The traditional medicines of Persia, Mesopotamia, India, Greece, and Rome were greatly influenced by the medicine of ancient Arabia (Abu-Rabia, 2015).

Due to the spreading of Islam over the territory of Near East, many medical books have been translated by Muslim scientists from Persian and Sanskrit languages into Arabic. Many Muslim physicians and scholars established the basis of herbal medicine in the Near East. The most famous ancient medical reference is *Qanoon f’il tibb* (in English: *Canon of Medicine*) authored by the famous Parisian physician Avicenna. It contained a comprising description of numerous frequently used medicinal plants (Ghazanfar, 2011). Between the 7th and 14th century, Muslim physicians such as Rhazes, Abulcasis, Avicenna, and Ibn al-Baitar diagnosed cancer and realized that a cure is only possible, if the cancer was identified at its earliest stage (Zaid et al., 2011).

Avicenna (980–1037 AD) suggested: “When cancer starts, it may be possible to keep it as it is, so that it will not increase and keep it non-ulcerated. It may happen sometimes that the stating cancer may be cured. But when it is advanced, verily will not” (Zaid et al., 2011; Ahmad et al., 2016). He described four methods to treat cancer: (1) total arrest, which was regarded as difficult; (2) preventing progress; (3) preventing ulceration; and (4) cure and treatment of the ulceration (Zaid et al., 2011). Avicenna advised that cancer medications should not be of much strength, since strong medications develop carcinogenic effects by themselves. Therefore, the right medications are: “pure minerals like washed pure tutty mixed with oils like rose oil and the oil of yellow gillyflower mixed with it” (Zaid et al., 2011).

Ibn al-Baitar (1197–1248 AD) identified that *Cichorium intybus* has anticancer properties and could be used to treat neoplastic disorders (Saad et al., 2008; Zaid et al., 2011). The famous Muslim physician Al-Kindi from the 10th century described several medicinal plants such as *Commiphora myrrha* (Nees), *Curcuma longa* L., *Moringa peregrina* (Forssk.) Fiori, *Physalis alkekengi* L., *Polypodium vulgare* L., and *Vicia ervilia* (L.) Willd. for cancer treatment (Ben-Arye et al., 2012a).

On the other hand, the Muslim physicians suggested surgery in case the tumor was small and accessible and not close to major organs. In his book (*Qanoon f’il tibb*), Avicenna described one of the very early surgical treatments for cancer, and he noted: “The excision should be radical and that all diseased tissue should be removed, which included the use of amputation or the removal of veins running in the direction of the tumor … so that nothing of these will be left.” He also recommended the “use of cauterization for the area being treated if necessary” (Zaid et al., 2011).

Based medicines belong to complementary and alternative medicine (CAM). It was reported that 35.9% of cancer patients were either past or present users of CAM, where the herbal-based medicines were the most commonly used form of CAM (Molassiotis et al., 2005; Olaku and White, 2011; Ahmad et al., 2016).

As many other nations, the people in Near East have been using a wide variety of medicinal herbs in their indigenous ethnopharmacological systems of traditional medicine. The most frequently reported medicinal plants used by cancer patients of the Near East countries with their methods of application are listed in **Table 3**.

**Table 3:** The most commonly used medicinal plants among Near East cancer patients.

Scientific name	Family	Part used	Methods of use	Location	Reference
*Allium cepa* L.	Amaryllidaceae	BU	Decoction, juice	PA, IS	Said et al., 2002; Jaradat et al., 2016
*Arbutus andrachne* L.	Ericaceae	FR	Decoction, syrup	PA, IS	Said et al., 2002; Jaradat et al., 2016
*Arum dioscoridis* Sibth et Sm.	Araceae	LE	Decoction	JO, PA	Hudaib et al., 2008; Jaradat et al., 2016
*Arum maculatum* L.	Araceae	LE, BU	Decoction	SY	Alachkar et al., 2011
*Arum palaestinum* Boiss.	Araceae	LE	Decoction	JO, PA, IS	Said et al., 2002; Hudaib et al., 2008, Jaradat et al., 2016
*Bongardia chrysogonum* (L.) Spach	Berberidaceae	TU	Decoction	JO	Assaf et al., 2013
*Calotropis procera* (Aiton)	Apocynaceae	AP	Decoction	AR, PA	Norton et al., 2009; Said et al., 2014, Jaradat et al., 2016
*Capparis spinosa* L.	Capparaceae	FR, L	Cooked, decoction	PA, SY, AR	Alachkar et al., 2011; Jaradat et al., 2016
*Centaurea hyalolepis* Boiss.	Compositae	FL, BA	Decoction	LE	Baydoun et al., 2015
*Clematis flammula* L.	Ranunculaceae	FL	Decoction	LE	Baydoun et al., 2015
*Colchicum autumnale* L.	Colchicaceae	WP	Powder	SY	Alachkar et al., 2011
*Crataegus azarolus* L.	Rosaceae	SE, FL, FR	Infusion, decoction	LE, IS	Baydoun et al., 2015
*Crocus sativus* L.	Iridaceae	FL	Infusion	IS	Said et al., 2002
*Cyclamen coum*	Primulaceae	FL, FR	Decoction	LE	Baydoun et al., 2015
*Ecballium elaterium* L.	Cucurbitaceae	WP or TU	Juice	PA, SY	Alachkar et al., 2011; Jaradat et al., 2016
*Ephedra alata* Decne.	Ephedraceae	WP	Decoction	PA	Saganuwan, 2010
*Euphorbia peplus* L.	Euphorbiaceae	Milky juice	Milky juice	AR	Rizk and El-Ghazaly, 1995
*Fagonia indica* L.	Zygophyllaceae	AP	Decoction	AR	Said et al., 2014
*Inula viscosa* (L.) Aiton	Asteraceae	FL	Decoction	JO, PA	Afifi-Yazar et al., 2011; Jaradat et al., 2016
*Nigella sativa* L.	Ranunculaceae	SE	Oil	AR	Alachkar et al., 2011
*Nerium oleander* L.	Apocynaceae	WP	Cream	PA	Jaradat et al., 2016
*Ononis viscosa* ssp. *sicula* (Guss.)	Leguminosae	WP	Decoction	PA	Jaradat et al., 2016
*Onopordum cynarocephalum* Boiss.	Compositae	FR	Prepared as tea	SY	Alachkar et al., 2011
*Phoenix dactylifera* L.	Arecaceae	FR	Decoction, juice	IR	Sadeghi et al., 2014
*Plantago lanceolata* L.	Plantaginaceae	LE, FL	Boiled	SY	Alachkar et al., 2011
*Pulicaria crispa* Forssk.	Asteraceae	AP	–	AR	Graham et al., 2000
Punica granatum L.	Lythraceae	FR	Syrup	JO, PA	Said et al., 2002
*Quercus calliprinos* Decne.	Fagaceae	FR, BA	Decoction	JO, PA,	Afifi-Yazar et al., 2011; Jaradat et al., 2016
*Raphanus raphanistrum* L.	Brassicaceae	LE, FR	Juice	SY	Afifi-Yazar et al., 2011; Jaradat et al., 2016
*Rhazya stricta* Decne.	Apocynaceae	AP, seeds	Decoction	AR	Phondani et al., 2016
*Ricinus communis* L.	Euphorbiaceae	SE	Oil	AR	Saganuwan, 2010
*Ruta graveolens* L.	Rutaceae	LE	Oil	AR	Saganuwan, 2010
*Sarcopoterium spinosum* L.	Rosaceae	RO	Decoction	JO	Hudaib et al., 2008
*Sinapis arvensis* L.	Cruciferae	LE, SE, RO	Sprouted seeds	SY	Alachkar et al., 2011
*Teucrium polium* L.	Lamiaceae	SE	Prepared as tea	SY	Alachkar et al., 2011
*Trifolium stellatum* L.	Leguminosae	AP	Prepared as tea	SY	Alachkar et al., 2011
*Urtica pilulifera* L.	Urticaceae	LE	Decoction	IS	Said et al., 2002
*Urtica dioica* L.	Urticaceae	LE, SE	Decoction	TU, IR	Malak et al., 2009; Yildiz et al., 2013
*Urtica urens* L.	Urticaceae	LE, SE	Decoction	JO, PA	Hudaib et al., 2008; Jaradat et al., 2016
*Varthemia iphionoides* Boiss.	Compositae	LE, FL	Cooked, decocted	JO	Assaf et al., 2013
*Viscum cruciatum* Sieber ex Boiss.	Santalaceae	LE	Decoction	JO, PA	Assaf et al., 2013; Jaradat et al., 2016
*Vitex agnus-castus* L.	Verbenaceae	FR, LE, FL, SE	Decoction, syrup	IS	Bachrach, 2012
*Withania somnifera* L.	Solanaceae	LE, FL	Decoction	JO, PA	Al-Qura’n, 2009; Jaradat et al., 2016
*Zhumeria majdae* Rech.	Lamiaceae	WP	Applied topically	IR	Zargari, 1992; Graham et al., 2000
*Ziziphus spina-christi* L.	Rhamnaceae	LE, ST	Infusion	JO, PA, IS	Said et al., 2002; Hudaib et al., 2008, Jaradat et al., 2016


**AP, aerial parts; BA, barks; BU, bulbs; FL, flowers; FR, fruits; LE, leaves; RO, roots; SE, seeds; ST, stems; TU, tubers; WP, whole plant. AR, Arabian Peninsula; JO, Jordan; PA, Palestinian Authority; LE, Lebanon; IR, Iran; IS, Israel; SY, Syria; TU, Turkey*.*

For this reason, comprehensive screenings on traditional medicinal plants and their chemical constituents for prevention and treatment of cancer raised considerable interest all over the world (Newman and Cragg, 2007; Olaku and White, 2011; Kuete and Efferth, 2015; Ahmad et al., 2016).

Traditional herbal medicine belongs to the leading CAM modality among patients in Near East countries (Uysal et al., 2016; Zarshenas et al., 2016; Sobeh et al., 2017). In the study conducted by Ben-Arye et al. (2012b) from six countries (Israel, Palestinian Authority, Egypt, Jordan, Morocco, and Turkey), it was shown that there is a high preference to use CAM and herbal medicine among both pediatric and adult cancer patients. Typical CAM-based recipes in these countries include honey for mucositis prevention in cases of head and neck cancer, wheat grass juice in the case of advanced breast cancer, kefir and yogurt in the case of colorectal cancer, and tomato lycopene supplementation in the case of colon cancer and others. HESA is a mixture composed of plant and marine materials, including *Penaeus latisculatus* (King Prawn), *Carum carvi*, and *Apium graveolens* is used to improve quality of life in colon and breast cancer patients (Ben-Arye et al., 2012a).

### Ethnopharmacology of the Arabian Peninsula

A survey conducted by Jazieh et al. (2012) revealed that 90% of the Saudi Arabian cancer patients used various types of CAM, e.g., 88% of these patients used non-dietary supplements and 85.2% used dietary supplements, including the holy water known as “*Zam Zam* water,” which is described in the Old Testament of the Bible and *Qur’ãn* and geographically springing from the barren desert surrounding Mekka as well as honey and black seed (*Nigella sativa*), which are recited in the *Qur’ãn*. Suleiman (2014) reported that *Acacia* plants are used among adult patients in Riyadh as anticancer agent. The aerial part of *Pulicaria crispa* is used for vaginal tumors (Graham et al., 2000).

Among various types of CAM used in Qatar for cancer care, herbal medicine was the most familiar one to oncology practitioners (Hassan, 2015). The milky juice of *Euphorbia peplus* is used for the treatment of cancer in Qatar (Rizk and El-Ghazaly, 1995). *Rhazya stricta* is Decne, which is native to the arid desert environment zones of the Arabian peninsula, Iran, Iraq, Afghanistan, Pakistan, and India (Boulos et al., 1994; Collenette, 1998) is among the most common and important used plants in the Arabian Peninsula for cancer treatment (**Table 3**) (Said et al., 2014; Phondani et al., 2016).

### Ethnopharmacology of Egypt, Israel, Jordan, Lebanon, Palestinian Territory, and Syria

The vast majority of cancer patients in Israel and Jordan use CAM. In Lebanon, CAM accounts for 15% among pediatric cancer patients. No data are available on the use of CAM among cancer patients in Egypt and Syria (Ben-Arye, 2014). Herbal medicine is the leading CAM modality among patients in the Palestinian territory, and 60.9% of cancer patients use medicinal plants for cancer treatment (Ali-Shtayeh et al., 2011). In Jordan, 35.5% of the cancer patients use herbal medicine (Afifi et al., 2010).

Since honey, olive oil, black seeds, and dates were specifically mentioned in the Holy *Qur’ãn*, they are frequently used as food supplements by cancer patients. It was also reported that 81.3% of cancer patients used the holy water *Zam Zam* either by drinking and bathing in it, and 24.4% used herbs (Akhu-Zaheya and Alkhasawneh, 2012). Afifi et al. (2010) reported that 73.3% of interviewed Jordanian cancer patients preferred infusions of crude herbal extracts, while 6.8% used the herbs as tablets or capsules and 19.8% used products prepared by herbalists, e.g., plant extract mixtures with honey or soaked in olive oil. Diverse reports described the medicinal plants of Jordanian flora, which are traditionally recommended for cancer treatment. Afifi-Yazar et al. (2011) listed 27 indigenous Jordanian medicinal plant species for cancer treatment with their methods of preparation and active constituents. Among these plants (**Table 3**), decoctions of the leaves of *Arum dioscoridis* Sibth et Sm., *Arum hygrophilum* Boiss., *Arum palaestinum* Boiss, *Globularia arabica* L., and *Platanus orientalis* L., are used for cancer treatment (Hudaib et al., 2008; Afifi-Yazar et al., 2011). These plants were listed in the Jordan Red List as endangered species (Taifour and El-Oqlah, 2015).

The herbalists, traditional practitioners, and healers of villages in the Palestinian territory used decoctions, infusions, and syrups of about 72 medical plants belonging to 44 families mainly for the treatment of lung cancer, but also liver, skin, colon, and breast cancers. The most frequently used remedies were decoctions of *Ephedra alata*, *A. dioscoridis*, and *A. palaestinum* (Jaradat et al., 2016). A survey conducted by Said et al. (2002) on medicinal herbs used in Israel, the Golan Heights and the West Bank region revealed that the decoctions of several plants species are used against various forms of cancer. Furthermore, Bachrach (2012) reported that *Vitex agnus-castus* and *Withania somnifera* are used in the folk medicine of Israel against cancer. Interestingly, *A. dioscoridis*, *A. hygrophilum*, *A. palaestinum* are indigenous species in the flora of Israel, Jordan, Lebanon, Palestinian territory, and Syria, while *V. agnus-castus* is native to the Mediterranean and Central Asian countries, including the countries of the Near East (Hanelt, 2001).

### Ethnopharmacology of Iran and Turkey

The traditional medicine of Iran rooted in the Persian civilization and was largely influenced by the Arabic Muslim civilization during the golden age of the Medieval-Islamic Empire. Iranian medical practitioners integrated various Middle Eastern medical systems with their own experiences and the result is now known as Iranian traditional medicine (ITM) (Naghibi et al., 2014a,b).

Most of the ITM authors explained cancer development by a similar etiology. They also pointed out that cancer is hard to cure or even incurable. On the other hand, they introduced different therapeutic approaches for cancer therapy, including pharmacotherapy, surgery, and cauterization (Naghibi et al., 2014b). Iranian medical scholars used 99 medicinal plants and various preparations thereof to manage cancer (Naghibi et al., 2014a). Between the 8th and 14th century, the Muslim physicians from Iran, described 43 medicinal plants for cancer treatment of and relieving its complications (Emami et al., 2012). *Artemisia campestris*, *Artemisia vulgaris*, *Asplenium adiantum-nigrum*, *Capparis* spp., *Equisetum* spp., *Euphorbia* spp., *Hypericum* spp., *Iris* spp., *Lycium lanceolatum*, *Lycium afrum*, *Matricaria recutita*, *Sambucus nigra*, *Sambucus ebulus*, *Tanacetum parthenium*, *Tripleurospermum disciforme*, *V. agnus-castus*, *Vitex negundo*, *Vitex pseudo-negundo*, *Urtica dioica*, *Urtica pillulifera*, and *Urtica urens* were among the medicinal plants described in the ITM Pharmacopoeia to cure cancer and cancer-like diseases (Naghibi et al., 2014a).

Nowadays, the preference to use CAM among Iranian cancer patients is still high and reached up to 35%. Prayer and spiritual healing were the most common CAM methods (Montazeri et al., 2007). However, only a few reports described the medicinal plants that are used in ITM to cure cancer. Sadeghi et al. (2014) reported that decoctions as well as the juice of fruit and seeds of palm date are taken by habitants of the Saravan region, Baluchistan in Iran for cancer treatment. The entire plant of *Zhumeria majdae* is topically applied on the cancer lesions (Zargari, 1992; Graham et al., 2000).

In Turkey, the prevalence to use CAM was about 36–52%. The patients in Northwestern Turkey preferred to take herbs as CAM after their diagnosis with breast cancer (Gulluoglu et al., 2008), and 41% of cancer patients in East Turkey apply alternative herbal treatments (Gözüm et al., 2003). In West Turkey, 42% cancer patients practice at least one CAM methods, and the rate of those using herbal alternative medicine is 36.8% (Malak et al., 2009). Interestingly, the leaves and seeds of *U. dioica* and *U. urens* (nettles) were the most common herbal medications used by 70% of the Turkish cancer patients (Inanç et al., 2006; Malak et al., 2009; Yildiz et al., 2013).

## *In Vitro* Cytotoxicity Studies of Medicinal Plants From the Near East Toward Cancer Cells

Owing to the fact that a vast majority of clinically approved and established anticancer drugs are derived from natural sources (Newman and Cragg, 2007), the US-American National Cancer Institute screened more than 35,000 plant samples with 114,000 extracts for their anticancer activity (Sithranga Boopathy and Kathiresan, 2010). In the past years, large screenings of medicinal plants from Asia and Africa have been performed (Efferth et al., 2007; Kuete et al., 2016, 2017; Mbaveng et al., 2017). Nevertheless, basic and clinical research aiming to study the cytotoxic activity of medicinal plants of the Near East to combat cancer is still very limited (Ben-Arye, 2014).

However, in the past decade, scholars from Saudi Arabia, United Arab Emirates, Oman, and Yemen raised interest on the *in vitro* cytotoxic activity of medicinal plants from the Arabian peninsula (Mothana et al., 2007, 2011; Almehdar et al., 2012; Ali et al., 2014; Said et al., 2014; Baeshen et al., 2015; Chhetri et al., 2015; Khasawneh et al., 2015; Ahmad et al., 2016; Al-Muniri and Hossain, 2017), while no data about cytotoxic activity of medicinal plants and other ethnopharmacological practices used in Kuwait, Iraq, and Qatar have been recorded. Ahmad et al. (2016) reported results of *in vitro* and *in vivo* cytotoxic activity, inhibition of cell proliferation, and cycle cell arrest of extracts of 42 medicinal plants used in Muslim and Arabic folk medicine for cancer therapy.

In Jordan, Lebanon, and the Palestinian territory, the published literature focused on studying herbal medicine and other ethnopharmacological sources that are used by local inhabitants for cancer treatment (Ali-Shtayeh et al., 2008, 2011; Afifi-Yazar et al., 2011; Jaradat et al., 2016). Some studies conducted by Jordanian and Palestinian researchers, focused on the *in vitro* cytotoxic activity of indigenous plants of the Jordanian and Palestinian flora (Abuharfeil et al., 2000; Alali et al., 2006; Fiore et al., 2006; Abu-Dahab and Afifi, 2007; Kaileh et al., 2007; Al-Kalaldeh et al., 2010; Talib and Mahasneh, 2010; Zihlif et al., 2012; Kasabri et al., 2014; Abu-Darwish et al., 2016; Al-Tamimi et al., 2016). In Lebanon and Syria, research focused on the *in vitro* cytotoxic activity of endemic or native medicinal plants of the Lebanese flora (El-Najjar et al., 2008; Abdel-Massih et al., 2010; Deeb et al., 2010; Jomaa et al., 2012; Saab et al., 2012; Tohme et al., 2013). In Egypt, some articles reported the cytotoxic activity of medicinal plants assayed by *in vitro* experiments (El-Shemy et al., 2003; Nassr-Allah et al., 2009; Aboul-Enein et al., 2012; Kuete et al., 2012; El-Seedi et al., 2013; Moustafa et al., 2014).

*In vitro* studies on medicinal herbs were the main research topics conducted by Iranian researchers (Amirghofran et al., 2005; Feizzadeh et al., 2008; Rajabalian, 2008; Tavakkol-Afshari et al., 2008; Varamini et al., 2009).

In contrast, the information on conducted clinical studies, investigating the clinical efficacy of medicinal plants from Near East is still very limited (Ben-Arye, 2014).

### Medicinal Plants of the Arabian Peninsula

Studies on medicinal plants used in the Arabian Peninsula region illuminated their *in vitro* activity, indicating their potential as sources for anticancer drug development (Ahmad et al., 2016). Cucurbitacin E glucoside and cucurbitacin I glucoside isolated from the fruits of *Citrullus colocynthis* L. used in the folk medicine of Saudi Arabia and other countries of Near East for cancer treatment (Hussain et al., 2014) exhibited cytotoxicity toward HepG2 hepatoma cells with IC_50_ values of 3.5 and 2.8 nmol/mL, respectively (Ayyad et al., 2012).

Almehdar et al. (2012) screened 40 medicinal plants used in the folk medicine of Saudi Arabia for their cytotoxic activity against breast cancer (MCF7), hepatocellular carcinoma (HepG2), HFB4 (normal melanocytes), and cervix cancer (HeLa) cell lines. The methanol extract of *Hypoestes forskaolii* was the most active against HFB4 with IC_50_ value 4.18 μg/mL, while the methanol extract of *Adenium obesum* which is native to south and east of Africa and found wildly in Oman, Saudi Arabia, and Yemen (Win et al., 2012) was the most cytotoxic extract against HeLa cells with an IC_50_ value 6.9 μg/mL. The methanol extract of *Capparis tomentosa* Lam. was the most potent extract against MCF7 with an IC_50_ value 9.05 μg/mL. In another study conducted by Mothana et al. (2011), the cytotoxic activity screening of methanol and water extracts of 33 medicinal plants used in Yemeni traditional medicine for treatment several ailments including cancer against epithelial 5637 and breast cancer MCF7 cell lines revealed that the methanol extracts of *H. forskaolii* was the most cytotoxic one against tested cells with IC_50_ values of 14.3 and 32.1 μg/mL, respectively. This study showed that methanol extracts of tested plants had significantly higher cytotoxicity than their corresponding water extracts and the epithelial 5637 cells were generally more sensitive than MCF7 cells.

*Leptadenia pyrotechnica* (Forssk.) Decne. is native plant to the Gulf countries and used in the folk medicine of the Arabian Peninsula for treatment of several diseases including cancer (Norton et al., 2009). The 80% ethanolic extract of as well as its fractions (*n*-hexane, ethyl acetate, *n*-butanol and water) were evaluated for cytotoxicity against colon cancer cell lines (HCT116 wild type and HCT116 p53^-/-^ knockout). The hexane fraction of *L. pyrotechnica* exhibited the highest cytotoxic activity against both cell lines and decreased cell viability in a dose- and time-dependent manner (Khasawneh et al., 2015).

*Rhazya stricta* Decne. which is used in the traditional medicine of Arabian Peninsula in the form of water decoction (**Table 3**) for treatment of cancer, has been studied *in vitro* for its cytotoxic activity against hepatocellular carcinoma (HepG2) and colon cancer cells (CaCo). In comparison with the water extract of *R. stricta*, the ethanol extract of *R. stricta* showed cytotoxic activity against HepG2 and CaCo cells with IC_50_ values of 25 and 35 μg/mL, respectively (El-Awady et al., 2015). Other studies demonstrated the cytotoxic activity of *R. stricta* and its active component rhazinilam toward various cell lines similar to the used control drug paclitaxel (Gu and Zakarian, 2010; Baeshen et al., 2015).

The chloroform and ethyl acetate extracts obtained from aerial part of *R. stricta* grown in Oman and tested for their *in vivo* cytotoxicity by a brine shrimp assay revealed significant cytotoxic activity with LC_50_ values 18.1 and 13.9 μg/mL, respectively. Extracts with the same solvents obtained from latex of *Calotropis procera*, which is used also by Omani and Arabian Peninsula folk medicine for cancer treatment (**Table 3**), for treating wounds, pain, scorpion stings, and for strengthening muscles affected by paralysis (Ghazanfar, 2011; Norton et al., 2009) have been also tested by the brine shrimp assay and exhibited cytotoxic activity with LC_50_ values 3.0 and 8.2 μg/mL, respectively (Said et al., 2014).

Methanol extracts of 24 medicinal plants of the Yemeni flora have been screened *in vitro* for their cytotoxic activity against panel of cancer cell lines, including lung cancer cell lines (A-427 and LCLC-103H), urinary bladder carcinoma (5637 and RT-112) and breast cancer (MCF7) lines. The highest toxicities on all tumor cell lines have been observed by methanol extracts of *Dendrosicyos socotrana*, *Withanina aduensis*, *Withania riebeckii*, *Dracaena cinnabari*, and *Buxus hildebrandtii* with IC_50_ values ranging between 0.40 and 1.47, 0.30 and 4.30, 0.29 and 3.78, 2.59 and 5.54, and 0.32 and 15.1 mg/mL, respectively (Mothana et al., 2007).

On the other hand, some aromatic medicinal plants from Yemen and their essential oils have been screened for their cytotoxic activity (Chhetri et al., 2015). The essential oil of *Pulicaria jauberti* which is distributed in southern Saudi Arabia and northern Yemen, exhibited an significant cytotoxic activity *in vitro* against MCF7 and HepG2 cells with IC_50_ values of 3.8 and 5.1 μg/mL, respectively (Fawzy et al., 2013; Chhetri, 2015), while another species from Yemen, *Pulicaria undulata*, which also native to the other countries of Arabian Peninsula and used in their traditional medicine (Norton et al., 2009) showed moderate *in vitro* cytotoxicity toward MCF7 cells with an IC_50_ value of 64.6 μg/mL (Ali et al., 2012).

### Medicinal Plants of Egypt, Israel, Jordan, Lebanon, Palestinian Territory, and Syria

In the past decade, many studies reported the *in vitro* cytotoxic activity of medicinal plants originated from Egypt, Israel, Jordan, Lebanon, Palestinian territory, and Syria (**Table 4**). El-Seedi et al. (2013) screened the cytotoxic activity of 61 Egyptian medicinal plants originated from the Sinai Desert. The methanol extracts of *Nerium oleander* which is traditionally used in cream form for treatment of skin cancer (Jaradat et al., 2016) and *Pulicaria undulate* were cytotoxic against human U-937 GTB lymphoma cells.

**Table 4:** Medicinal plants of the Near East with most considerable cytotoxic activity *in vitro.*

Scientific name	Origin	Extract	Cell line	Activity	Reference
*Astragalus spinosus*		Ethanol	EACC	Inhibited completely (100%) of cell growth	
(Forssk.) Muschl.					
*Atriplex* spp.					
*Solanum nigrum* L.					
*Arum palaestinum* Boiss.	EG	Ethanol	EACC	Inhibited 97.29% of cell growth.	Aboul-Enein et al., 2012
*Cistanche phelypaea* L.		Water	EACC	Inhibited 100% of cell growth.	
*Solenostemma argel*				Inhibited 95% of cell growth	
*Cassia italica* Mill.				Inhibited 92% of cell growth.	
*Cakile maritima* Scop.				Inhibited 90.78% of cell growth.	
*Ferula hermonis* Boiss.	EG	CH_2_CL_2_	HMPC, MCF7, CCRF-CEM	IC_50_: <20 μg/mL	Kuete et al., 2012
*Erysimum corinthium* Boiss.	EG	70% Ethanol	HepG2	IC_50_: 2.5 μg/mL	Ateya et al., 2014
*Origanum syriacum* L.	JO	Ethanol	MCF7	IC_50_: 6.4 μg/mL	Al-Kalaldeh et al., 2010
*Citrus limon* L.	SY	Essential oils	LIM1863	IC_50_: ranging from 5.75 to 7.92 μg/mL	Jomaa et al., 2012
*Calotropis procera* (Aiton)	IR	Methanol	NCF-7, HepG2, A-549, and MDBK cells	IC_50_: ranging from 1.9 to 12.16 μg/mL	Esmaeili et al., 2014
*Urtica dioica* L.		Ethanol	T47D	IC_50_: 46.14 μg/mL	Kashani et al., 2014
*Taverniera spartea* DC.		Methanol	By brine shrimp lethality assay	LC_50_: 0.34 μg/mL	Khalighi-Sigaroodi et al., 2012
*Tephrosia persica* Boiss.				LC_50_: 2.43 μg/mL	
*Hypoestes forskaolii* (Vahl) R.Br.					
*Withania somnifera* L.					
*Solanum glabratum* Dunal var. sepicula	SA	Methanol	MCF7 HepG2 HeLa	IC_50_: below 20 μg/mL	Almehdar et al., 2012
*Adenium obesum* Forssk. *Pistacia vera* L. *Eulophia petersii* Rchb.f.					
*Colchicum sanguicolle* K.M. Perss	TU	Methanol	HeLa cells	IC_50_: 2 ± 0.02 μg/mL	Artun et al., 2015
*Pulicaria jauberti* (syn. *Pulicaria orientalis* Jaub. & Spach)	YE		MCF7 HepG2	IC_50_: ranging from 3.8 to 5.1 μg/mL	Fawzy et al., 2013


**EACC, Ehrlich ascites carcinoma cells; HMPC, human MIA PaCa-2 pancreatic cancer cells; MCF, MCF7 breast cancer cells; CCRF-CEM, CCRF-CEM leukemia cells; LIM1863, Doxorubicin-resistant colorectal cancer cell line; HepG2, hepatocellular carcinoma; HeLa, cervix cancer; T47D, invasive ductal carcinoma. EG, Egypt; JO, Jordan; IR, Iran; SA, Saudi Arabia; SY, Syria; TU, Turkey; YE, Yemen*.*

*Arum palaestinum*, which is traditionally used for cancer treatment in several countries of the Middle East, was among medicinal plants of the Egyptian flora that showed superior *in vitro* cytotoxic activity. It inhibited the growth of Ehrlich ascites carcinoma cells (EACC) by 97.29% (Aboul-Enein et al., 2012).

The 95% ethanol extract of *Diplotaxis harra* originated from South and North of Sinai, Egypt exhibited cytotoxic activity against HCT116, HepG2, and MCF7 cell lines with IC_50_ values 4.65, 12.60, and 17.90 μg/mL, respectively. The flavonoids isolated from this extract including quercetin, quercetin 3-*O*-β-glucoside, isorhamnetin 7-*O*-β-glucoside, apigenin 3-*O*-β-rhamnoside, and kaempferol 3-*O*-β-glucoside also showed *in vitro* cytotoxic activity against the same cell lines with IC_50_ values of 20.1, 24.3, 22.8, 23.4, and 41.9 μg/mL, respectively (Mohammed et al., 2011).

Among 16 medicinal plants originated from Egypt, the dichloromethane crude extract of *Ferula hermonis* exhibited remarkable *in vitro* cytotoxic activity against human MIAPaCa-2 pancreatic cancer cells, MCF7 breast cancer cells, CCRF-CEM leukemia cells, and their multidrug-resistant subline, CEM/ADR5000 with IC_50_ values below 20 μg/mL (Kuete et al., 2012).

The essential oil of leaves and berries of *Juniperus phoenicea* grown in Sinai, Egypt exhibited high cytotoxic activities against brain and lung, liver and breast human cell lines with IC_50_ values of 0.6, 0.7, and 0.8 μg/mL, respectively (El-Sawi et al., 2007).

Based on their traditional use as anticancer agents, the 50% ethanol extracts of 17 plants from Israel have been tested for their cytotoxic activity against various human cancer cell lines including LNCaP prostate adenocarcinoma, Colo 205 colon carcinoma, Hec-1A endometrial adenocarcinoma, OVCAR-3 ovarian carcinoma, HepG2 hepatocellular carcinoma, MCF7 breast carcinoma, 293 embryonic kidney adenocarcinoma, Karpas 299 T-cell non-Hodgkin’s lymphoma, A494 alveolar basal epithelial adenocarcinoma, SU-DHL-1 anaplastic large cell lymphoma, YC and OSTRA normal EBV-transformed lymphoblasts, HUT-102T-cell lymphoma, and T24P urinary bladder carcinoma. Among all tested plants, the extracts of *Urtica membranacea*, *Artemisia monosperma*, and *Origanum dayi* at concentrations up to 3 mg/mL exhibited superior time- and concentration-dependent cytotoxic activity against all cancer cell lines tested, but not against normal human cells (Solowey et al., 2014). The ethanol extract of chemotype of *Varthemia iphionoides* originated from Israel exhibited remarkable cytotoxic activity against HL-60 leukemia cells, and this activity was stronger than that on other cell lines including SKOV3 ovarian carcinoma cells, BG melanoma cells, and A549 lung cancer cells (Yarmolinsky et al., 2015).

*Asphodelus microcarpus*, *Ecballium elaterium*, *Eryngium creticum*, *Mercurialis annua*, *Pistacia lentiscus*, *Rhamnus alaternus*, *Teucrium polium*, and *Urtica pilulifera* are used in traditional Arab medicine in Israel and the Palestinian territory. Water extracts of these plants have been investigated for their effects on mitochondrial respiration and cell membrane integrity in PC12 and HepG2 cells. Except for the *E. elaterium* extract, none of the other extracts inhibited mitochondrial respiration in these cells. The water extract of *E. elaterium* significantly caused concentration-dependent inhibition of mitochondrial respiration in a concentration range of 0.1–1 mg/mL in HepG2 cells (Ljubuncic et al., 2005). Interestingly, the fruit juice of *E. elaterium* is used in Palestinian and Syrian folk medicine to treat liver and throat cancer (Alachkar et al., 2011; Jaradat et al., 2016).

Few studies have focused on the cytotoxic activity of Jordanian medicinal plants. Assaf et al. (2013) screened the cytotoxic activity of the methanol extracts of *M. annua*, *Bongardia chrysogonum*, and *Viscum cruciatum* against Burkitt’s lymphoma and U266-IgE producing myeloma cells. Only the *V. cruciatum* extract exhibited selective cytotoxic activity against Burkitt’s lymphoma with an IC_50_ value of 14.21 μg/mL. The decoction of *V. cruciatum* is used in Jordanian and Palestinian folk medicine against esophageal cancer (Assaf et al., 2013; Jaradat et al., 2016).

The analysis of 44 extracts obtained from 16 medicinal plants from Jordan in HepG2, MCF7, and Vero cells revealed that 20 of these extracts obtained from *Ononis hirta*, *Ononis sicula*, *Inula viscosa*, *Salvia pinardi*, and *Verbascum sinaiticum* showed moderate cytotoxic activity against one or more of the cell lines tested. The methanol fraction of the flowers of *I. viscosa* were the most active against MCF7 cells with an IC_50_ value of 15.78 μg/mL (Talib and Mahasneh, 2010). The decoction of the flowers of *I. viscosa* is used in Jordanian and Palestinian folk medicine against kidney and bladder cancer (Afifi-Yazar et al., 2011; Jaradat et al., 2016).

The colchicinoid *N*,*N*-dimethyl-*N*-deacetyl-(-)-cornigerine isolated from *Colchicum crocifolium* growing in the Jordanian desert exhibited remarkable cytotoxicity against MCF7 breast carcinoma, NCI-H460 large cell lung carcinoma and SF-268 astrocytoma cells with IC_50_ values of 0.515, 1.00, and 1.77 μg/mL, respectively (Alali et al., 2010).

The *in vivo* administration of the aqueous extracts of *N. sativa* seeds and *Allium sativum* bulbs, which are popular in Jordan folk medicine to treat cancer, augmented splenic NK cells with values of 62.3 ± 6.4 and 52.6 ± 5.4% cytotoxicity, respectively (Abuharfeil et al., 2001).

*Withania somnifera*, *Psidium guajava*, *Laurus nobilis*, and *Salvia fruticosa* are among medicinal plants that are used in folk medicine of Palestinian authority and Jordan for cancer treatment. However, from 24 indigenous Palestinian plants screened for their cytotoxic activity against the murine L929sA fibro sarcoma cells and two human breast cancer cell lines (MDA-MB231 and MCF7), only *W. somnifera*, *P. guajava*, *L. nobilis*, and *S. fruticosa* exhibited weak cytotoxic activity. The IC_50_ values of the extract of *W. somnifera* on L929sA and MCF7 cells were 150 and 60 μg/mL, respectively, while the IC_50_ value of the *P. guajava* extract on MCF7 cells was 55 μg/mL (Kaileh et al., 2007).

Some studies reported the cytotoxic activities of plants originated from Syria and Lebanon. Farhan et al. (2013) reported that the ethanol and aqueous extracts of the leaves and stems of *Trigonella berythea* grown in south Lebanon inhibited the growth of MCF7 and U937 cell lines by more than 60%. The IC_50_ values ranged from 29.46 to 61.54 μg/mL. The essential oils obtained from *Cedrus libani*, *Pinus pinea*, *Juniperus oxycedrus*, and *Juniperus excelsa* grown in Lebanon showed remarkable cytotoxic activity toward drug-sensitive human CCRF-CEM leukemia cells and their multidrug-resistant P-glycoprotein-expressing subline CEM/ADR5000, which did not exhibit considerable cross-resistance toward these extracts with degrees of resistance of less than twofold, although CEM/ADR5000 cells revealed high resistance toward doxorubicin as a control drug (Saab et al., 2012). The essential oils of two *Salvia* species (*S. bracteata* and *S. rubifolia*) used in traditional Lebanese medicine exhibited *in vitro* cytotoxic activities against human M14 melanoma cells at concentrations that were non-toxic to normal cells. The oil of *S. rubifolia* was significantly (*p* < 0.001) more active as the essential oil of *S. bracteata* (Cardile et al., 2009).

The essential oils obtained from the peel of *Citrus limon* collected from four different locations in Syria inhibited cells viability of the doxorubicin-resistant colorectal cancer cell line LIM1863 with IC_50_ values from 5.75 to 7.92 μg/mL. The cytotoxicity of Syrian lemon peel essential oils has been correlated to its abundant monoterpene limonene which is known to induce apoptosis and phase 1 and 2 carcinogen-metabolizing enzymes to prevent the interaction of chemical carcinogens with DNA (Jomaa et al., 2012).

### Medicinal Plants of Iran and Turkey

Compared to other countries in Near East, the cytotoxicity of medicinal plants from Iran and Turkey has been more intensively investigated. Naghibi et al. (2014b) investigated the cytotoxicity of methanol extracts from 19 plant species The authors used MCF7, HepG2, A-549, and HT29 cancer cells. Although these plants have been described in ITM to manage cancers, all of them showed no or only weak cytotoxic activity with IC_50_ values below 100 μg/mL. However, the methanol extract of *Tanacetum polycephalum* was more cytotoxic with IC_50_ values of 28.3, 53.0, and 43.3 μg/mL in MCF7, A-549, and HT-29 cell lines, respectively.

The milky latex and the leaves of *C. procera* is used by Iranian traditional healers to treat several diseases including cancer (Oloumi, 2014). Among 27 medicinal plants from the southern provinces of Iran, the methanol extract of the aerial parts of *C. procera* showed the highest cytotoxicity in a cell line panel consisting of MCF7, HepG2, A-549, and MDBK cells with IC_50_ values ranging from 1.9 to 12.16 μg/mL (Esmaeili et al., 2014).

Cancer patients from Near East also use decoctions of the aerial parts of *U. dioica* for cancer treatment (Malak et al., 2009; Yildiz et al., 2013; Naghibi et al., 2014a). However, among the ethanol extracts obtained from Iranian medicinal plants (*Alyssum homolocarpom*, *U. dioica*, *C. intybus*), which have been tested against HT-29, Caco-2 T47D cancer cell lines as well as Swiss mouse embryo fibroblasts (3T3), only the extract of the aerial parts of *U. dioica* revealed cytotoxicity (IC_50_ value in T47D cells: 46.14 μg/mL) (Kashani et al., 2014).

By using an *in vivo* brine shrimp lethality assay, the methanol extracts of 23 plant species belonging to Leguminosae family and originated from Iran have been screened. Testing crude methanol extracts of native plants from Iranian flora revealed that *Taverniera spartea* and the endemic plant *Tephrosia persica* showed high cytotoxic activity with IC_50_ values of 0.34 and 2.43 μg/mL, respectively (Khalighi-Sigaroodi et al., 2012).

The essential oils obtained from the peel of *Citrus limon*, *C. medica*, and *Camellia sinensis* collected in Iran exhibited cytotoxic activity against MCF7 and HeLa cells. The IC_50_ values were in a range between 0.5 and 17 μg/mL (Monajemi et al., 2010). These findings are in agreement with Jomaa et al. (2012), who showed that the essential oil of *C. limon* collected in Syria was cytotoxic against human LIM1863 colorectal carcinoma cells.

Ozkan et al. (2016) reported on some native and endemic Turkish plants with cytotoxic activity against several types of cancer cell lines. Artun et al. (2015) screened the methanol extracts of native and endemic Turkish plants, including *Colchicum sanguicolle* (endemic), *Crataegus microphylla*, *Teucrium sandrasicum* (endemic), *Centaurea nerimaniae* (endemic), *Centaurea antiochia* var. *praealta* (endemic), *Olea europaea*, *Cotinus coggygria*, *Hypericum kotschyanum* (endemic), *Nepeta italica*, *Stachys cretica* ssp. *vacillans*, *Scorzonera tomentosa* (endemic), *Origanum sipyleum* (endemic), *Rosa damascene*, and *Salvia hypargeia* (endemic), for their cytotoxicity against Vero and HeLa cells. Among them, six plants were cytotoxic against Vero cells and 11 against HeLa cells. The methanol extract of the cormus of *C. sanguicolle* was the most cytotoxic against HeLa cells (IC_50_ values of 2 ± 0.02 μg/mL).

Despite *Bellis perennis* L. and *Convolvulus galaticus* Rostan ex Choisy (Grizzle bindweed), an endemic plant of Turkish flora, are used Turkish folk medicine for treatment of several ailments including cancer, the methanol extract of the aerial parts of *B. perennis* exhibited only weak cytotoxicity against MCF7 cells (IC_50_: 71.6 μg/mL). The dichloromethane extract of the aerial parts of *C. galaticus* revealed some activity against HepG2/C3A cells (IC_50_: 57.3 μg/mL) (Karakas et al., 2015).

KL-21, a commercial Turkish product contains extracts from *Achillea millefolium*, *Acorus calamus*, *Cichorium endivia*, *C. longa*, *Equisetum arvense*, *Fumaria officinalis*, *Juniperus communis*, *Hypericum perforatum*, *Lavandula stoechas*, *Melissa officinalis*, *N. sativa*, *Peganum harmala*, *Rosmarinus officinalis*, *Silybum marianum*, *Solidago virgaurea*, *Taraxacum officinale*, *Thymus vulgaris*, *U. dioica*, *Valeriana officinalis*, *Viscum album*, and *Zingiber officinale.* KL-21 decreased the viability of 232B4 chronic lymphocytic leukemia cells in a dose- and time-dependent manner, while it did not affect the viability of normal BEAS-2B epithelial cells up to 100 μg/mL (Gökbulut et al., 2015).

The essential oil of *Origanum acutidens* (Hand.-Mazz.), an endemic plant originated from East Anatolia was weakly cytotoxic against HT-29 and HeLa cells at concentrations of 50 and 100 μg/mL. The cytotoxic activity of the essential oil of *O. acutidens* has been attributed to the carvacrol, which was the main component of this oil (Oke-Altuntas and Demirtas, 2017).

The seeds of *N. sativa* have been reported in Islamic, Arabic, and Turkish folk medicines to treat various diseases. The essential oil of *N. sativa* inhibited the proliferation of human malignant melanoma cells. This activity has been attributed to the main components of this oil, such as thymoquinone, anethole, trans-isoeugenol, carvacrol, and α-thujene (Zaid et al., 2011; Duran et al., 2016).

The medicinal plants from Near East with considerable *in vitro* cytotoxic activity are listed in **Table 4**.

## Mechanisms of Actions of Medicinal Plants From Near East Toward Cancer Cells

Medicinal plants represent an indispensable resource of pharmacologically active compounds with complex molecular structures. The cytotoxic and antitumor activity of these compounds results from various mechanisms, such as their activity on cytoskeletal proteins, which play a main role in cell division, inhibition of DNA topoisomerases, anti-protease or antioxidant activity and many others. Furthermore, medicinal plants and their biologically active compounds are useful to fight cancer by strengthening the immune system, decreasing side effects of synthetic anticancer drugs, overcoming resistance to chemo- and radiotherapy, exerting synergistic drug interactions in combination with other drugs, etc. (Mantle and Wilkins, 2005; Bachrach, 2012; Singh et al., 2016; Efferth, 2017; Nankar and Doble, 2017; Nankar et al., 2017; Wagner and Efferth, 2017; Zacchino et al., 2017a,b). Biologically active compounds from medicinal plants frequently target tumor cells by several mechanisms, resulting in the inhibition of carcinogenesis,, angiogenesis, oxidative stress, and induction of cell cycle arrest, extrinsic and intrinsic apoptosis, autophagy, or differentiation (Efferth and Koch, 2011; Chen et al., 2013; Millimouno et al., 2014; Singh et al., 2016; Aung et al., 2017).

Ahmad et al. (2016) reviewed medicinal plants used in Arabic and Islamic medicine to fight cancer. For example, the seeds of *A. graveolens* that are used in folk medicine of Saudi Arabia showed antiproliferative activity and induced apoptosis in human BGC-823 stomach cancer cells after cell cycle arrest in the S-phase (Gao et al., 2011; Ahmad et al., 2016). Furthermore, the methanol extract of the aerial parts of *Artemisia absinthium* demonstrated antiproliferative effects on human breast cancer cells and triggered apoptosis by modulation of Bcl-2 family proteins and the MEK/ERK pathway (Shafi et al., 2012). The cucurbitacin glucosides isolated from *C. colocynthis* grown widely in Saudi Arabia caused both cell cycle arrest and apoptosis in breast cancer cells (Mukherjee and Patil, 2012; Hussain et al., 2014). The ethyl acetate fraction obtained from *A. palaestinum* suppressed proliferation of MCF7 with an IC_50_ value of 59.09 ± 4.1 μg/mL and leukemia cells with an IC_50_ of 53.1 ± 2.9 μg/mL, but not HepG2 cells (El-Desouky et al., 2007). Furthermore, thymoquinone as a main active compound of *N. sativa* seeds induced apoptosis and inhibited proliferation of colorectal, breast, ovarian, and human pancreatic adenocarcinoma, lung carcinoma, human osteosarcoma, uterine sarcoma, neoplastic keratinocytes, and fibrosarcoma cell lines (Shoieb et al., 2003; Gali-Muhtasib et al., 2004; Yi et al., 2008; Ahmad et al., 2016).

Oleuropein, a phenolic compound isolated from olive oil inhibited proliferation and induced apoptosis in MCF7 cells. This compound also arrested cell cycle progression at the G1 phase (Han et al., 2009; Zaid et al., 2011; Ahmad et al., 2016).

The cytotoxic activity of the ethyl acetate extract of *Crataegus azarolus* leaves against human HCT-116 and HT-29 colorectal cancer cells was associated with DNA fragmentation, loss of mitochondrial potential, and cleavage of poly (ADP-ribose) polymerase (PARP) and caspase-8 (Mustapha et al., 2016). The authors suggested the involvement of the extrinsic pathway of apoptosis, which was associated with enhanced p21 expression, but not p53 activation.

*Leptadenia pyrotechnica* (Arabic: *Markh*) is widely used in the Arabic Gulf region to treat cancer. The hexane fraction obtained from the 80% ethanol extract of the aerial parts of *L. pyrotechnica* was very weakly cytotoxic against colon cancer cells (IC_50_: 100 μg/mL). It induced p53-dependent apoptosis in human colon cancer cells through intrinsic as well as extrinsic pathways. Western blot analyses showed that the hexane fraction activated caspases and led to an upregulation of Bax and downregulation of Bcl-2 in a time-dependent manner in p53^-/-^ HCT 116 (Khasawneh et al., 2015).

Analyzing the cell morphological changes, DNA fragmentation and using an annexin V assay, showed that the 95% ethanol extract of *Lavandula dentate* originated from Saudi Arabia induced apoptosis in MCF7 cells (Ali et al., 2013).

Zaid et al. (2011) reported on the activities of herbs used in Arab-Islamic medicine. *N. sativa* and it is active ingredient thymoquinone revealed superior cytotoxicity against various cancer cell lines both in cell culture and animal models. It blocked tumor angiogenesis in a human xenograft prostate cancer model in mice (Kaseb et al., 2007). Thymoquinone also induced apoptosis in xenograft tumors. In a comparable manner, thymoquinone also inhibited the growth of colon tumors implanted into nude mice without side effects on normal tissues (Gali-Muhtasib et al., 2004; Yi et al., 2008).

*Punica granatum* and its juice were reported in Arabic and Muslim medicine to fight cancer (Zaid et al., 2011). Indeed, pomegranate juice inhibited the growth of colon, lung, and breast cancer cell lines *in vitro*. The growth of PC3 cells was inhibited by modulation of the cyclin kinase inhibitor-cyclin-dependent kinase machinery (Malik and Mukhtar, 2006; Zaid et al., 2011).

Ben-Arye et al. (2012a) presented 44 medicinal plants used in the folk medicine of the Middle East region to fight cancer. *Boswellia carteri*, *C. sinensis*, *Dracaena draco*, *Helleborus niger*, *N. sativa*, *P. lentiscus*, and *Salvia* sp. induced apoptosis in a panel of different cell lines derived from melanoma, fibrosarcoma, prostate cancer, myeloid leukemia, lymphoma, cervical cancer, and colon cancer. These authors also showed that *Glycyrrhiza glabra* induced cell cycle arrest in PC-3 cells. *C. colocynthis*, *C. myrrha*, *Crocus sativus*, *Cyperus rotundus*, *Urtica* sp., and *V. album* had an antiproliferative effect toward various cancer cell lines too.

Animal experiments with Egyptian desert plants showed that the hot water extract of *Solenostemma argel* Nect. (family: Asclepiadaceae), whose leaves are widely used in traditional medicine as laxative, antipyretic, and antispasmodic remedy, significantly reduced the growth of EACC and delayed the death of female Swiss albino mice transplanted with EACC by 29 days. DNA fragmentation assays confirmed that the cytotoxicity of *S. argel* extract was due to the induction of apoptosis (Nassr-Allah et al., 2009).

The ethanol extracts of *U. membranacea*, *A. monosperma*, and *O. dayi* originated from Israel was cytotoxic against various cancer cell lines (Solowey et al., 2014). These extracts also induced apoptotic cell death of Hec-1A endometrium cancer cells.

Decoctions of *I. viscosa* and *Ononis viscosa* are used in the folk medicine of Jordan and Palestinian Authority (**Table 3**) to treat several types of cancer (Afifi-Yazar et al., 2011; Jaradat et al., 2016). Among 20 polar and non-polar extracts obtained from various medicinal plants of Jordan, the methanol extracts obtained from the aerial parts of *O. hirta* and the flowers of *I. viscosa* showed the most significant antiproliferative activity, which was attributed due to their ability to induce apoptosis (Talib and Mahasneh, 2010).

The cytotoxic activity of *S. bracteata* Banks et Sol. and *S. rubifolia* Boiss. which are used in Lebanese folk medicine for treatment of several diseases including cancer, toward human M14 melanoma cells was due to their induction of apoptotic cell death (Cardile et al., 2009). The antiproliferative activity of the essential oil of *Satureja montana* in comparison with oils of other aromatic Lebanese plants such as *Lavandula officinalis*, *Mentha arvensis*, *T. vulgaris*, and *Salvia officinalis* was due to the ability of *S. montana* to induce erythroid differentiation of human leukemic K562 cells (Lampronti et al., 2006).

Itani et al. (2008) determined the cellular and molecular mechanisms of the cytotoxic effects of linalyl acetate, terpineol and camphor isolated from Lebanese sage (*Salvia libanotica*) against two isogenic colon cancer cell lines (HCT-116 p53^+/+^ and p53^-/-^). The combination of these three compounds synergistically inhibited the growth of these colon tumor cell lines, but did not reveal any growth-inhibitory effect on normal FHs74Int human intestinal cells. All compounds tested induced apoptosis in p53^+/+^ and p53^-/-^ cells. The induction of apoptosis in p53^+/+^ cells by these three components was associated with increased Bax/Bcl-2 and phosphorylated p53/non-phosphorylated p53 ratios, loss of mitochondrial membrane potential, cleavage and activation of caspase-3, and cytochrome *c* release. On the other hand, a lesser pronounced disruption of mitochondrial membrane potential was observed in p53^-/-^ cells, and caspase activation was not involved in cell death induction. At the same time, linalyl acetate, terpineol, and camphor induced PARP cleavage in both tested cell lines. Furthermore, the authors concluded that apoptosis in p53^+/+^ cells was mediated by mitochondrial-mediated, caspase-dependent cell death pathway, while in p53^-/-^ cells cell death happened mainly in a caspase-independent manner.

Among 36 medicinal plants used in Iranian folk to treat cancer, 24 induced apoptosis (Asadi-Samani et al., 2016). The cytotoxic activity of *Avicennia marina*, *A. absinthium*, *Z. officinale*, *P. alkekengi*, *Achillea wilhelmsii*, *Mentha pulegium*, fruits of *A. sativum*, seeds of *N. sativa*, and shoots of *Ferula gummosa* was associated with their ability to induce apoptosis in the cancer cell lines tested. Roots and shoots of *A. absinthium*, roots of *G. glabra*, leaves and fruits of *O. europaea*, leaves of *C. sinensis*, the stigma of *C. sativus* and the resin of *Boswellia serrata* inhibited proliferation. Furthermore, the authors reported that *Ferula assa-foetida* inhibited mutagenesis. The cytotoxic activity of *F. assa-foetida* was attributed to its main coumarin component, umbelliprenin, which induced apoptosis in chronic lymphocytic leukemia cell lines in a dose- and time-dependent manner, where interleukin-4 had no effect on apoptosis induction (Ziai et al., 2012). The caspase-3 and annexin-V/propidium iodide assays confirmed that the methanol extract of *Hypericum scabrum* originated from Iran induced apoptosis in MCF7 cells (Hamzeloo-Moghadam et al., 2015).

The induction of apoptosis in MCF7 cells by the methanol extract of *T. polycephalum* ssp. *argyrophyllum* grown in Iran was due to the activation of caspase 3 (Naghibi et al., 2014b). Shahneh et al. (2013) observed that a methanol extract of the indigenous Iranian plant *Echinophora platyloba* inhibited the proliferation of fibrosarcoma cells in a time- and dose-dependent manner via induction of apoptosis.

In comparison to Iranian flora, less investigations on the elucidation of mechanisms of actions from Turkish medicinal plants have been conducted. Tokgun et al. (2012) reported that methanol extracts of *C. galaticus*, *Crocus antalyensis*, and *Lilium candidum* originated from Turkey were cytotoxic toward MCF7 cells with IC_50_ values of 0.32, 0.72, and 1.06 μg/mL. These plants induced apoptosis in MCF7 cells. Western blot analyses confirmed that these plants induced apoptosis by cellular p53 accumulation.

The induction of apoptosis in HL-60 cells by a methanol extract of the Western Turkish endemic plant *Scutellaria orientalis* ssp. *carica* plant was caused by genotoxic stress as shown by phosphorylation of the core histone γ-H2AX (Özmen et al., 2010). This was followed by caspase-3 activation and PARP cleavage.

The commercial natural product preparation KL-21 exhibited cytotoxic activity against chronic lymphocytic leukemia cells. The ability of KL-21 to induce apoptosis was correlated with the activation of a mitochondrial/caspase-3-dependent pathway and the inhibition of cell cycle progression through the G0/G1 phase (Gökbulut et al., 2015).

## Clinical Anticancer Trials With Medicinal Plants From the Near East

It is apparent from the published data that there is a far-reaching lack of clinical trials with cancer patients investigating the clinical activity of herbal preparations usually applied in the Near East. Iranian researchers conducted randomized single-blinded clinical trials on cancer patients, who received various herbal medicines or other CAM preparations. In a randomized single blind clinical trial conducted by Motallebnejad et al. (2008), Iranian patients with head and neck cancer were treated with radiotherapy. The patients have been divided into two groups of each 20 patients. The first group was instructed to take 20 mL honey 15 min before radiation therapy, then again at intervals of 15 min and 6 h after radiation. In the second group (control group), the patients were instructed to take 20 mL saline before and after radiation. The authors observed a significant reduction in oropharyngeal mucositis among the patients, who received honey in comparison with the control group (*p* < 0.001).

In another study conducted by Ahmadi et al. (2010), 30 Iranian patients with end-stage cancers and liver metastases have been treated for 3 months with 50 mg/kg/day of a natural drug preparation known as HESA-A. The mean Karnofsky Performance Scale scores of the patients increased from 48 ± 14.36 to 78.42 ± 15.37 after 12 weeks of treatment, indicating an improvement of quality of life. Furthermore, 90.4% of the patients were alive for at least 12 weeks.

Other clinical studies also focused on the role of CAM practices to improve the life quality of cancer patients or their ability to reduce the side effects of cancer chemotherapy (Safarinejad, 2005; Panahi et al., 2012; Jafari et al., 2013).

## Conclusion and Perspectives

Folk medicine of the Near East populations has a long-lasting, rich tradition, since Persian, Mesopotamian, Greece, and Roman civilizations had greatly influenced medicine of the ancient Near East, followed later by the influences of the Arabic and Islamic civilization, which spread over its territory. Still, nowadays, most societies of the Near East region rely on herbal medicine and CAM to treat diseases. Except for Iran, where prayer and spiritual healing was the most common CAM method against cancer, traditional herbal medicine is the leading practice among cancer patients of the other countries of the Near East.

**FIGURE 1:**
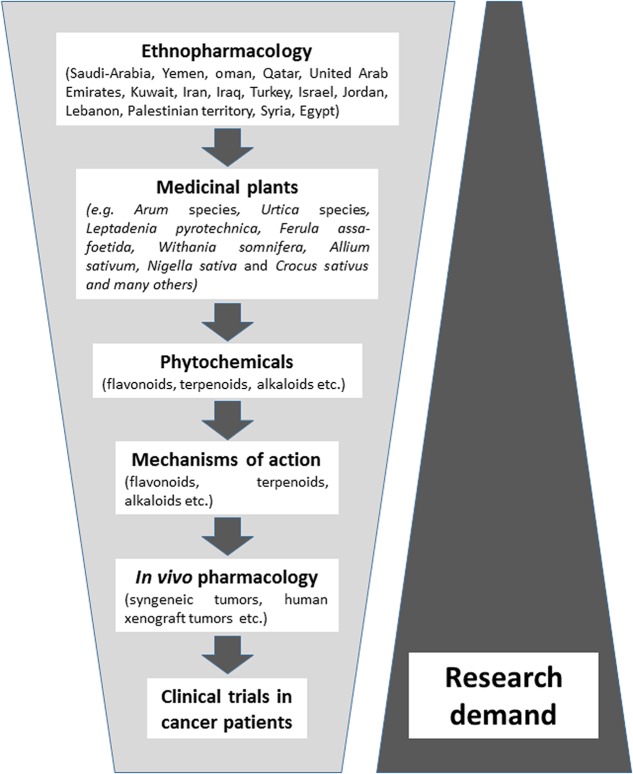
Synopsis of the phytotherapeutic drug development process with medicinal plants from the Near East.

The majority of published literature represents ethnopharmacological surveys and their use in folk medicine of the Near East, followed by *in vitro* studies on the cytotoxic activity of these plants against cancer cell lines. Considerably less attention has been paid as of yet on the phytochemistry of bioactive compounds in these plants and the determination of cellular and molecular mechanisms of action. There is still a large gap of information on *in vivo* experiments and clinical studies in cancer patients using plant preparations or isolated phytochemicals. Without the doubt, here is a great demand for future studies (**Figure 1**).

Summarizing the conducted surveys on anticancer ethnopharmacology of various countries of the Near East, the following plant species belong to the mostly used ones for cancer treatment, various *Arum* species (*A. dioscoridis*, *A. palaestinum*, *A. maculatum*, and *A. dioscoridis*), *C. procera*, various *Artemisia* species (*A. absinthium*, *A. campestris*, *A. monosperma*, *A. vulgare*), *N. sativa*, *C. colocynthis*, *P. crispa*, *W. somnifera*, and various *Urtica* species (*U. dioica*, *U. membranacea*, *U. pilulifera*). Interestingly, *C. sanguicolle*, *O. acutidens*, *S. orientalis* ssp. *carica*, *T. persica*, which are endemic to the Near East flora also were cytotoxic activity by several mechanisms of action.

The flora and traditional medicine of the Near East provide a rich source of medicinal plants for the development of novel treatment strategies. Further phytochemical and mechanistic investigations as well as *in vivo* experimentation and clinical investigations are required to integrate treatment practices of traditional medicine into conventional oncology for the sake of cancer patients not only in Near East, but everywhere on this globe.

## Author Contributions

MA-D and TE conceived and designed the study. MA-D involved in collecting and acquisition of literature data, analysis and interpretation of collected literature data, and drafting and submission of the manuscript. TE critically reviewed the manuscript.

## Conflict of Interest Statement

The authors declare that the research was conducted in the absence of any commercial or financial relationships that could be construed as a potential conflict of interest.
